# Prevalence and impact of comorbid PTSD, c-PTSD and EUPD on symptom severity in functional neurological disorder: protocol for a systematic review and meta-analysis

**DOI:** 10.1136/bmjopen-2025-101122

**Published:** 2025-10-16

**Authors:** Sian Davies, Damir Rafi, Raphael Rifkin-Zybutz, Simon Heyland, Ferozkhan Jadhakhan

**Affiliations:** 1 University of Birmingham College of Medical and Dental Sciences, Birmingham, UK; 2 Birmingham and Solihull Mental Health NHS Foundation Trust, Birmingham, UK; 3 South London & Maudsley NHS Foundation Trust, London, UK; 4 King’s College London, London, UK; 5 Specialist Psychotherapies Service, Birmingham and Solihull Mental Health NHS Foundation Trust, Birmingham, UK; 6 Birmingham City University, Birmingham, UK

**Keywords:** Functional Neurological Disorder, Personality disorders, Stress, Psychological, MENTAL HEALTH

## Abstract

**Abstract:**

**Introduction:**

Previous trauma and adverse life experiences have been hypothesised to be aetiological factors for functional neurological disorder (FND) leading to the hypothesis of a ‘trauma-subtype’ of FND. Individuals who have experienced prior abuse are more likely to develop FND than healthy controls. Post-traumatic stress disorder (PTSD) and personality disorders have been described to be comorbid with FND at varying prevalence rates. Complex PTSD (c-PTSD) and emotionally unstable personality disorder (EUPD) have clinical similarities with PTSD and trauma is a common aetiological factor in the development of all three conditions. There is some research exploring the resemblance of personality traits in populations diagnosed with FND and EUPD compared with controls. However, it remains unclear what the current prevalence rates are of PTSD, c-PTSD and EUPD in populations diagnosed with FND. Understanding the overlap between these trauma-based comorbid psychiatric diagnoses with FND will hopefully inform better and more comprehensive trauma-informed treatment strategies. The aims of the systematic review are therefore to (1) establish the prevalence of PTSD, c-PTSD and EUPD in adults diagnosed with FND and (2) compare core FND symptom severity between adults with FND alone and those with comorbid PTSD, c-PTSD and EUPD.

**Methods and analysis:**

This systematic review will assess the prevalence of PTSD, c-PTSD and EUPD in FND patients aged over 18, diagnosed using standardised questionnaires or clinical interviews as per international diagnostic criteria. Studies will be identified through comprehensive searches of databases including PsycINFO, PsycARTICLES, CINAHL, MEDLINE, EMBASE, Web of Science and Scopus, from May 1990 to May 2025. The review aims to estimate the prevalence of these conditions in FND, with findings presented as a narrative description discussing contributing factors, and a meta-analysis considered if heterogeneity is suitable.

**Ethics and dissemination:**

Ethical approval is not required since only data from existing studies will be used and no original data will be collected. Results will be disseminated at national and international academic conferences and in peer-reviewed publications. Any deviations from this protocol will be recorded and explained in the final report and updated on PROSPERO.

**PROSPERO registration number:**

CRD42024599112.

**STRENGTHS AND LIMITATIONS OF THIS STUDY**

- A strength of the systematic review is that it will include studies that detail the prevalence of clinician-diagnosed post-traumatic stress disorder (PTSD)/complex PTSD (c-PTSD)/emotionally unstable personality disorder (EUPD) in populations diagnosed with functional neurological disorder (FND).
- It will look at studies which explore the core FND symptom severity in those diagnosed with FND alone and comorbid PTSD/c-PTSD/EUPD.
- A comprehensive framework will be used to extract data. The findings will be summarised narratively after consideration of bias and quality of data.
- A weakness is that, due to large heterogeneity in presentation of FND, the review will only focus on the core symptoms rather than also exploring other associated symptoms, for example, fatigue and pain.
- It may not be possible to undertake a meta-analysis as results are likely to be highly heterogenous due to the variation of outcome measurements.

## Introduction

 Functional neurological disorder (FND) refers to the experience of neurological symptoms without recognised neurological disease.^1^ It is a common cause of chronic and disabling symptoms, which may be varied in nature and severity. Common symptoms include motor weakness and/or paralysis, sensory disturbances, seizures, speech or visual disturbance, and cognitive impairment. FND can be as distressing and debilitating as severe neurological disorders such as epilepsy or multiple sclerosis.^2^

It is estimated that around 16% of new outpatient neurological referrals are due to FND,^3^ and the diagnostic challenge is such that functional seizures, for example, are reported to have an average of 7 years between onset of initial symptoms and definitive diagnosis.^4^ Patients with FND are at high risk of inappropriate treatments, high levels of distress and disability, and iatrogenic harm.^5^ The healthcare costs associated with FND are also significant, with one systematic review discussing that accurate diagnosis could provide an avenue to reducing these costs.^6^

Psychiatric comorbidity is extremely common in sufferers of FND. It is estimated that the overall prevalence of comorbid psychiatric diagnoses ranges from 51% to 95%.^7^ Depression and anxiety are currently estimated to be most prevalent; however, some studies have been conducted showing a high prevalence of comorbid post-traumatic stress disorder (PTSD).^8^ Veterans of the US military have an especially high comorbid risk of PTSD, particularly those suffering from functional seizures.^9^

Recent studies have shown that maltreatment and traumatic life events are significantly more prevalent in FND sufferers than in controls.^10^ While trauma has long been hypothesised as a potential aetiological factor in the development of FND, the exact prevalence and nature of the interaction between trauma and FND are less clear. Some studies have looked into medically unexplained symptoms and found that poor attachment, including childhood neglect and abuse, can significantly shift how people experience and interpret physical symptoms in their later life.^11^

From a neurobiological perspective, brain imaging studies have provided some insight into how physical symptoms, such as those in FND, can develop after trauma. One functional MRI study, published in 2014, demonstrates enhanced connectivity between the amygdala and motor regions in patients with FND who had suffered adverse events.^12^ Literature also indicates that changes in emotional processing, including hyperactivation of limbic and motor systems, and altered interoception of bodily emotional responses, could contribute to the establishment of FND symptoms. Such changes may arise from early life adversity, trauma or neglect.^13^ Evidence also suggests that individuals with FND are more susceptible to dissociation than controls.^14^ Despite some recent advances in this field, more work is nonetheless required on elaborating on precisely how trauma can pre-dispose to FND, and how prevalent trauma and FND are.^15^

Computational neuroscience has more recently shed some light into the possible links between trauma-based disorders and FND.^16^ The concept of predictive processing posits that the brain continually adapts to new and complex environments by updating and refining predictions about the external world. Such predictions ultimately stem from past experiences and from overarching life beliefs. The brain’s predictions about a new situation ultimately influence interoception, exteroception and proprioception—thus impacting upon how our bodies feel, act and move.^16^ Research suggests that trauma can induce changes in brain networks—such as the salience and default mode networks, which can in turn impact upon the processing of new information.^17^ Aberrant predictive processing and elevation of biological stress markers are also thought to be linked to the development of FND, resulting in mismatches between environments, perceptions and interpretations.^18 19^

While trauma and FND appear to be linked, estimates of diagnosed comorbid PTSD in FND sufferers vary widely between studies, and there still exists a lack of understanding around the full interaction and relationship between the conditions. However, as has been discussed, proposed commonalities such as hyperactivation of limbic systems, aberrant predictive processing and stress reactivity mean that the link between FND and PTSD has become more established in recent years. This in turn, if established further, can result in more targeted treatments. Studies have already begun to be conducted attempting to use eye movement desensitisation and reprocessing (EMDR), traditionally a treatment used only in PTSD, for FND.^20^

Complex PTSD is a relatively newer diagnosis, appearing for the first time in the International Classification of Diseases (ICD)-11 classification. As in PTSD, past trauma is a necessary feature, and individuals suffering from complex PTSD often have sustained or multiple exposures to trauma.^21^ While trauma is not a necessary criterion to meet the diagnosis of emotionally unstable personality disorder (EUPD), childhood trauma is known to be extremely common in sufferers of the condition, with over 70% of patients known to have suffered from abuse or maltreatment.^22 23^ Complex PTSD and EUPD share many common features, with many complex PTSD sufferers exhibiting difficulties with self-organisation, affective dysregulation, feelings of worthlessness and disturbances in relationships. This marked overlap, alongside the high prevalence of trauma in EUPD alone, necessitates, in our view, that we include EUPD in this analysis.^24^ In addition, studies have identified shared personality traits between patients diagnosed with FND and EUPD compared to controls.^25 26^

The primary aim of this systematic review, therefore, is to ascertain the overall prevalence of comorbid trauma-based psychiatric diagnoses in FND. While previous studies have explored in detail the prevalence of other morbid conditions—especially depression and anxiety^27^—we aim to similarly investigate the prevalence of diagnoses that are based around trauma to provide more clarity in an area where reports vary.^28 29^ The diagnoses we will focus on are PTSD, complex PTSD and EUPD which share trauma as a common aetiological factor.^30^

Our secondary aim of this systematic review is to compare symptom severity and relapse rate between FND alone and those with comorbid PTSD, complex PTSD and EUPD. Understanding this can further help us to ascertain the nature of FND, potentially opening up new treatment pathways. As far as we are able to determine, there have not been previous systematic reviews exploring these specific aims.

Through this work, we hope to contribute positively to the ongoing discussion of whether FND is an alternative way for trauma to manifest, and how best to manage symptoms in those suffering from comorbid diagnoses. Contributing to this discussion and improving clarity in terms of diagnosis can, in the longer term, potentially improve patient outcomes and reduce associated healthcare costs.

### Aims

The systematic review will aim to (1) establish the prevalence of PTSD, c-PTSD and EUPD in adults diagnosed with FND and (2) compare core FND symptom severity between adults with FND alone and those with comorbid PTSD, c-PTSD and EUPD. Exploring this difference in symptom severity may allude to important associations between comorbid psychiatric conditions and the prognosis of FND.

## Methods and analysis

This review will be undertaken in line with the Preferred Reporting Items for Systematic Reviews and Meta-Analyses guidelines and was registered with the International Prospective Register of Systematic Reviews CRD42024599112.

### Population

The cases included in the review will be of adults aged 18 years or older who have a diagnosis of FND. Studies which include any participants less than 18 years old will be excluded. Authors may be contacted in order to provide raw data if this information is not described. The mean or median age (in years) and a description of the distribution (SD, range or IQR) will be detailed. In instances where the raw data cannot be obtained, the study will be excluded.

Recognised measures of PTSD, c-PTSD, EUPD and FND should be used in the studies.

For PTSD, this could involve using any of the following: a diagnosis based on a clinician-administered interview according to the Diagnostic and Statistical Manual of Mental Disorders (DSM)-III,^31^ DSM-IV-R,^32^ DSM V^33^ or ICD-9,^34^ ICD-10,^35^ ICD-11^36^ or a clinician-administered questionnaire such as the Clinician-Administered PTSD Scale for DSM-5 (CAPS-5).^37^

A recognised clinician-based diagnosis of c-PTSD should be used such as the ICD-11.

For EUPD, this could involve using a diagnosis based on a clinician-administered interview according to DSM-III, DSM-IV-R, DSM-V, ICD-9, ICD-10 or ICD-11 or through use of a diagnostic tool such as the Structured Clinical Interview for DSM–IV Personality Disorders (SCID–II).^38^

FND will be defined as per clinician-determined diagnosis, such as according to DSM-V, ICD-10 or ICD-11, and can include any subtype of FND. Data will be analysed for each subtype of FND if available. Studies will be excluded if they do not meet our accepted criteria for diagnoses as summarised in table 1.

**Table 1:** Eligible measures of diagnosis/symptom severity

Diagnosis/outcome domain	Examples of accepted measures
PTSD	DSM-III, DSM-IV-R, DSM V, ICD-9, ICD-10, ICD-11, CAPS-5
C-PTSD	ICD-11
EUPD	DSM-III, DSM-IV-R, DSM V, ICD-9, ICD-10, ICD-11, SCID–II
FND	DSM V, ICD-10, ICD-11
Core FND symptoms	CGI-I patient (±clinician, ±carer) Seizure frequency S-FMDRS PMDRS (for FMD)

CAPS-5, Clinician-Administered PTSD scale for DSM-5; CGI-I, Clinical Global Impression - improvement scale; C-PTSD, Complex Post Traumatic Stress Disorder; DSM, Diagnostic and Statistic Manual of Mental Disorders; EUPD, Emotionally Unstable Personality Disorder; FMD, Functional Movement Disorder; FND, Functional Neurological Disorder; ICD, International Statistical Classification of Diseases and Related Health Problems; PMDRS, Psychogenic Movement Disorders Rating Scale; PTSD, Post Traumatic Stress Disorder; SCID, Structured Clinical Interview for DSM; S-FMDRS, Simplified Functional Movement Disorders Rating Scale.

Unfortunately, there is no single measure identified to measure symptom severity across the range of FND symptoms in adults. We will focus on studies that measure severity of core FND symptoms (such as seizures, movement disorder, paralysis/weakness and mixed motor symptoms); however, we are aware that people with FND can experience a wide range of additional physical symptoms (eg, fatigue, pain).^39 40^ There is marked heterogeneity and variability of symptoms, which is often accompanied with pronounced discrepancy between objective measures and subjective experience of symptoms.^41^ In the absence of rigorously validated and widely endorsed FND-specific outcome measures, we will accept the recommendations which were devised by the Functional Neurological Disorder-Core Outcome Measures group which integrated research findings with expert opinion.^42^

The following core FND symptom measures will therefore be accepted: the Clinical Global Impression-Improvement Scale (CGI-I) and patient-reported seizure frequency which are the most consistently used measures of symptom change. The main advantage of the CGI-I is that it can be used across the full range of FND symptoms. The Psychogenic Movement Disorder Rating Scale and Simplified Functional Movement Disorders Rating Scale will be accepted for assessment of Functional movement disorders. The data will be analysed separately for each outcome measure used.

### Included study types

We will include observational studies which detail the prevalence of PTSD, c-PTSD, EUPD and FND using clinician-based diagnostic measures.

### Comparators

We will include studies which detail the prevalence of comorbid PTSD/c-PTSD/EUPD in populations with FND. We will also look at studies that show differences in symptom severity between patients with FND alone and with comorbid PTSD/c-PTSD/EUPD. Studies which do not include a control group of patients with a sole diagnosis of FND will be excluded.

### Outcomes

The primary outcome is the prevalence of comorbid PTSD, c-PTSD or EUPD in adults aged over 18 diagnosed with FND. Results of prevalence will be documented as a percentage, in order to standardise values across studies.

The secondary outcome is the symptom severity in those diagnosed with FND with/without a comorbid diagnosis of PTSD, c-PTSD or EUPD. We will extract and report on the symptom severity based on the type of assessment used.

### Study design

The review will include retrospective and prospective cohort studies, case-control, nested case-control and cross-sectional studies. Case studies, randomised controlled trials and qualitative studies will be excluded.

The studies will have had to have been published in peer-reviewed scientific journals. Grey literature will not be included due to absence of peer review of the data.

### Search strategy

The following databases will be searched from May 1990 to May 2025 and the search will be updated prior to manuscript submission: PsycINFO, PsycARTICLES, CINAHL, MEDLINE, EMBASE, Web of Science and Scopus. May 1990 has been chosen as only studies where diagnoses of FND have been made according to DSM-V, ICD-10 or ICD-11 will be eligible; DSM-V was published in 1993^43^ and the ICD-10 was first endorsed in May 1990.^44^ Only studies written in the English language will be included as there is unfortunately no capacity to undertake accurate translation of texts.

A customised search strategy was developed with the assistance of the Birmingham and Solihull Mental Health NHS Foundation Trust team (see box 1). A librarian was consulted and reviewed the search strategy.

Box 1 **Search strategy**

(PTSD OR “Post traumatic stress disorder” OR “complex PTSD” OR cPTSD OR “complex post traumatic stress disorder” OR EUPD OR “Emotionally unstable personality disorder” OR “borderline personality disorder” AND (“functional neurological disorder*” OR “functional movement disorder*” OR “functional motor disorder*” OR “functional seizure*” OR “psychogenic non-epileptic seizure*” OR “psychogenic seizure*” OR “conversion disorder*” OR NEAD OR “non-epileptic attack disorder*” OR “non epileptic*” OR nonepileptic* OR “dissociative seizure*” OR “functional sensory” OR “functional tremor*” OR “functional disorder*” OR “psychogenic movement*” OR “psychogenic speech”))

### Preparing for eligibility screening

Prior to commencement of screening, the Rayyan^45^ reference management tool will be used to assemble search results into a library. Any duplicates will be identified and removed.

### Study selection

Data from the literature search will be uploaded to Rayyan software, a web-based systematic review programme. Each study will be independently screened by two reviewers who will read the paper titles and abstracts in order to select articles for full-text screening. Studies will be screened by SD, DR, RaB, RoB, JD and DZ. Any duplicates will be removed. Eligibility of remaining full-text articles will be further screened and evaluated by two reviewers using an adapted Hayden *et al*’s framework^46^ (see online supplemental appendix 1). This eligibility criteria will ensure consistency during the process and is summarised in table 2. If there are any disagreements between the two reviewers undertaking study selection, a third adjudicator will be invited to join further discussion to resolve any disputes.

**Table 2:** Eligibility criteria

Study design	Retrospective and prospective cohort studies Case-control Nested case-control Cross-sectional studies
Study characteristics	Written in English Language Study identified via electronic database search Full-text article available
Participants	Adults aged (≥18 years) with a diagnosis of FND and comorbid PTSD/c-PTSD/EUPD Recognised diagnostic measures should be used, as outlined in table 1
Outcome measures	Data on prevalence rates of PTSD/c-PTSD/EUPD comorbidity in FND populations as compared with control Recognised measures of core FND symptom severity should be used, as outlined in table 1

c-PTSD, Complex Post Traumatic Stress Disorder; EUPD, Emotionally Unstable Personality Disorder; FND, Functional Neurological Disorder; PTSD, Post Traumatic Stress Disorder.

A Preferred Reporting Items for Systematic Review and Meta-Analysis Protocol (PRISMA-P) flow diagram will be used and the review will be carried out according to PRISMA-P guidelines.^47^ Excluded studies, along with reasons for exclusion, will be described in a PRISMA-P flow diagram which we will provide.

### Quality assessment

Our comprehensive database search and careful screening of relevant studies by two independent reviewers using the forms outlined in online supplemental appendix 1 will aid in ensuring the quality of included studies. Further quality assessment of full-text articles that meet eligibility criteria will be undertaken independently by two reviewers using an adapted version of the Joanna Briggs Institute’s (JBI) Critical appraisal checklist for studies reporting prevalence data (online supplemental appendix 2).^48^ A third reviewer will be included in the discussion if there is any disagreement in regard to the inclusion of a study. The adapted JBI Critical appraisal checklist will first be piloted on known papers to determine its feasibility. The quality assessment of studies will include reviewing the sampling method, sample size, data analysis and statistical analysis. In addition, studies will be assessed for whether valid methods were used for identification and measurement of the conditions. The quality of each study will be provided in a table and then critically appraised to discuss its impact on the results.

The JBI Critical appraisal checklist has been chosen based on its consistency with the Grading of Recommendations, Assessment, Development and Evaluation approach to assessment of uncertainty.^49^ Risk of bias will be displayed as ‘yes’ indicating low risk, ‘no’ indicating high risk and ‘unclear’ for each domain (online supplemental appendix 2). If a meta-analysis can be undertaken, then a sensitivity analysis will be run to remove low-quality studies to see if this affects the results.

### Data extraction

The following data items will be extracted for each study: authors, year of publication, study location, study design, summary statistics and methods for statistical analysis. We will also record the presence and source of bias. Any disagreements will be discussed by two reviewers, involving a third reviewer if necessary. We will present agreement between reviewers via the Cohen’s kappa score, for abstract screening, full-text screening and data extraction separately.^50^

### Data analysis and synthesis

The primary outcome is the prevalence of PTSD/c-PTSD and EUPD in those diagnosed with FND. Prevalence rates will be extracted from the data available. If there are enough studies available (minimum two studies),^51^ we will calculate weighted estimates of prevalence within relevant subgroups; we will explore differences by study design and diagnostic criteria. Pooled prevalence estimates with 95% CIs will be calculated. We will provide a table summarising study characteristics in the results to help in the synthesis of evidence.

Heterogeneity of studies will be assessed using I^2^ statistics which will give a percentage of variability in the effect estimate due to heterogeneity rather than due to chance. I^2^ values of 25%, 50% and 75% are suggestive of low, moderate and high heterogeneity, respectively.^52^ The inverse variance method of DerSimonian and Laird will be used to estimate between-study heterogeneity in prevalence of PTSD/c-PTSD/EUPD in people diagnosed with FND.^53^ It is likely that a random effects model will be used to pool prevalence estimates as it is expected that there will be heterogeneity among the study results. If a meta-analysis can be undertaken, then we will run a sensitivity analysis to see how removal of low-quality studies affects prevalence estimates.

Secondary outcomes will look at the core symptom severity of those diagnosed with FND with and without comorbid PTSD/c-PTSD/EUPD. Due to the variability in outcome measures used to describe core FND symptom severity, it is likely that we will record absolute values rather than comparing differences in results across the varying types of symptoms. However, if enough studies (>2) use comparable symptom severity measures, we will pool study results using standardised mean differences as a summary effect measure. If there are enough data, then we may undertake a meta-regression to explore the relationship between symptom severity and the presence of comorbid PTSD/c-PTSD/EUPD.

Publication bias will be assessed by using Egger’s statistics with 95% CIs and examining the symmetry of an associated funnel plot.^54^ If Egger’s test is significant (p<0.05), the funnel plot is asymmetric, suggesting bias.

Any amendments to the protocol will be tracked and dated. The systematic review is likely to be finalised by June 2026.

### Patient and public involvement statement

Neither patients nor members of the public contributed to the creation of the systematic review protocol. The protocol was devised as there was a lack of previous systematic reviews or meta-analyses looking at the outcomes of interest: prevalence of PTSD/c-PTSD/EUPD in patients diagnosed with FND and the associated differences in symptom severity and healthcare costs.

## Discussion and implications of results

This systematic review will aim to present a synthesis of the best available evidence describing the prevalence of PTSD, complex PTSD and EUPD in patients who suffer from FND. The review will highlight the strengths and limitations identified in the literature. The review will provide a reliable estimation of the prevalence of comorbid PTSD, as well as some analysis of the difference in clinical presentation between sufferers of both conditions compared with solely FND. Through this, the review will hope to contribute to the active and ongoing discussion as to how best to manage and treat FND, and whether different strands of patients should be offered tailored, individualised care based on their presentation and comorbidities. The discussion around the use of EMDR for patients with comorbid PTSD, for instance, is one example of how understanding the heterogenous presentation of FND, its causes and comorbidities, can potentially guide and tailor future treatment. FND patients should be viewed holistically, tailoring treatment to their own unique, distinct presentation, rather than viewing them simply by a diagnosis, and we hope that this review can aid in this objective.

Moreover, recent studies have highlighted the substantial economic burden of FND^6 55^ and the fact that diagnostic uncertainty contributes significantly to these costs. By ascertaining the differences in outcomes between those with comorbid trauma-based diagnoses, targeted interventions could help to reduce healthcare costs in FND sufferers.

The overall hope is that this review can quantify the overlap of trauma and FND more succinctly and precisely, enabling more effective solutions to be found, while working within a constrained healthcare system.

### Ethics and dissemination

Ethical approval is not required since only data from existing studies will be used. Results will be disseminated at national and international conferences and in peer-reviewed publications.

## Supplementary material

10.1136/bmjopen-2025-101122 online supplemental appendix 1

10.1136/bmjopen-2025-101122 online supplemental appendix 2
