# Supplementary material for: Prevalence and impact of comorbid PTSD, c-PTSD and EUPD on symptom severity in functional neurological disorder: protocol for a systematic review and meta-analysis
Source: BMJ Open. 2025 Oct 16;15(10):e101122. doi: 10.1136/bmjopen-2025-101122 (PMC12530432; doi:10.1136/bmjopen-2025-101122)
Supplement: online supplemental appendix 1 [file bmjopen-15-10-s001.docx]

**Data extraction form adapted from the framework of Hayden et al.**

Eligibility criteria for the title and abstract screening phase

Inclusion criteria

| Study design | Assessment | Comment |
| --- | --- | --- |
| Is it:  [1] A cohort study (prospective or retrospective)  [2] A case-control or nested case-control  [3] A cross-sectional study | Yes  No  Unclear |  |
| Population | | |
| [1] Adults aged ≥18 years old  [2] Patients have a diagnosis of FND  NB: Please answer NO if mixed age population (i.e. some participants <18 years old) | Yes  No  Unclear |  |
| Comparators | | |
| Includes any of the following differences in:  [1] diagnostic rates  [2] core symptom severity  Between FND patients with and without co-morbid PTSD/ c-PTSD/ EUPD | Yes  No  Unclear |  |
| Outcome | | |
| Did the study report any of the following outcomes:  [1] Prevalence of PTSD/ C-PTSD/ EUPD in patients with FND  [2] core symptom severity between FND alone/ with co-morbid PTSD/ c-PTSD/ EUPD | Yes  No  Unclear |  |
| Final decision (please tick) | Include  Exclude  Unclear |  |

Exclusion criteria

| Reasons for exclusion study from review (please circle where appropriate) | |
| --- | --- |
| Study design | [1] Clinical trial  [2] Case study  [3] Qualitative study |
| Population | Age <18 |
| Intervention | [1] Randomised Clinical Trial |
| Comparator | Studies of patients with FND without PTSD/c-PTSD/ EUPD |
| Outcomes | Relevant outcomes not assessed:  [1] no diagnostic assessment of PTSD/c-PTSD/ EUPD to determine prevalence  [2] no assessment of core symptom severity |
| Other | Duplicate publication  Other (explain) |

| Eligibility criteria for Full Text | |
| --- | --- |
| Satisfaction of eligibility criteria above | Yes  No  Unclear |

Organisation:

| Organisational aspect | | Exclude: | Include: |
| --- | --- | --- | --- |
| Reviewer/date: | | Checked by: | |
| Author/ Year |  | | |
| Journal/ source |  | | |
| Country of origin |  | | |
| Publication type | Full text/ Abstract/ Book Chapter/ progress report/  Other – please specify | | |
| Fate | Decision: pending/ checked reference/ use for discussion/ exclude without listing/ exclude with listing  Other- please specify | | |
| Notes |  | | |

Data extraction template for full-text articles

| General study characteristics | |
| --- | --- |
| Location of study  Please specify country, type of healthcare setting/ system |  |
| Study Aims | Reported/ Not reported |
| Date of recruitment | From: to:  Median (range): #  Mean: # |
| Length of follow-up of outcome of interest + length of follow-up of study | From: to:  Median (range): #  Mean (standard deviation):# |
| Outcome definition | [1]PTSD/c-PTSD/EUPD  Median (range):#  Mean (standard deviation):#  [2]Symptom severity  Median (range):#  Mean (standard deviation):# |
| Outcome measurement | Did the study report measurements for any of the following outcomes:  [1] Prevalence of PTSD/c-PTSD/EUPD  -specify diagnostic measure used  (is it a clinical interview with diagnosis according to recognised diagnostic system)  [2] core FND symptom severity  -specify instrument and range (e.g.CGI-I)  -is it self-reported?  -is it clinician assessed? |
| Covariates/ confounders considered | [1] Did the study report measurements used to report  -patient characteristics and contexts associated with those diagnosed with PTSD/c-PTSD/EUPD in people with FND  *Please consider patient demographic (e.g. age, ethnicity, gender, homelessness) and clinical factors (physical co-morbidities)* |
| Relationship between outcome and relevant covariates/ confounders | Is the relationship statistically significant?  Yes/ No  If Yes:  OR/mean difference (95% confidence intervals):#  If No, offer reason:  Low powered or inconclusive study  A true negative study  Other reasons (please specify) |
| Power Calculation | Yes/No/Not reported  Calculated sample size:#  Sample size achieved: Yes/No |
| Funding | Unclear  Not reported  Please state where reported |
| Conflict of interest statement | Yes/No/Not reported |

| Observational study characteristics | |
| --- | --- |
| Sample size |  |
| Number of excluded patients |  |
| Recruitment method |  |
| Type of observational study | Cohort studies (prospective/ retrospective)  Case-control studies/ nested case-control  Cross-sectional studies |
| Age groups comparable | Yes/No  If No, please specify |
| Any confounders considered? | Yes/No  If Yes, specify which  If No, please specify |
| Analyses |  |
| Drop-outs stated | Yes/ No  If Yes: number in each group |

| Patient Characteristics | | | |
| --- | --- | --- | --- |
| Notes: Any relationship with outcomes?  Yes/No/Not reported  If Yes, please state statistical parameters and significance level where appropriate | Exposure (i.e. co-morbid PTSD/c-PTSD/EUPD) | Control  (i.e. sole diagnosis of FND) | Comments |
| Number of patients |  |  |  |
| Country |  |  |  |
| Age mean/ median (standard deviation/ range) |  |  |  |
| Ethnicity (Number, %) |  |  |  |
| Sex (Number, %) | Male:  Female: | Male:  Female: |  |
| Homelessness specified in study? |  |  |  |
| No of patients recruited |  |  |  |
| No of patients allocated |  |  |  |
| No of patients evaluated |  |  |  |
| No of drop-outs |  |  |  |
| Reasons for drop-out |  |  |  |
| Definition of PTSD/c-PTSD/EUPD/FND in the groups:  [1] via clinician based diagnosis  *Please specify* |  |  |  |
| Clinical status at recruitment:  [1] Comorbidities  Yes/No  *If yes, please state*  Number: |  |  |  |
| Adverse event?  Yes/No  *If Yes, please detail* |  |  |  |

Outcome details

*The following table can be copied for every relevant outcome assessed (please fill out fields only where applicable)*

| Outcome assessed | |
| --- | --- |
| Definition of each outcome |  |
| Time of assessment of each outcome |  |
| Timing of assessment |  |
| Length of follow up for each outcome |  |
| Method of measurement |  |
| No of patients evaluated for each outcome, as stated above |  |
| Confounding variables were considered (e.g. age, gender, ethnicity, homelessness, physical co-morbidities) |  |
| How were the confounding variables controlled? |  |

| Methodological quality summary | | | | | |
| --- | --- | --- | --- | --- | --- |
| Reviewer/ Date: | | | *Checked by:* | | |
| Contents | Yes | Partly | No | Unsure | Comments |
| Study participation |  |  |  |  |  |
| Study attrition |  |  |  |  |  |
| Measurement of outcome |  |  |  |  |  |
| Analytical Approach |  |  |  |  |  |
| Specify confounding variables measurements (e.g. age, gender, ethnicity, homelessness, physical comorbidities) |  | | | | |
| Specify method of controlling for confounding variables |  |  |  |  |  |
| Summary | Low risk of bias | | Moderate risk of bias | | High risk of bias |
| Remarks: |  | | | | |
