# Supplementary material for: Prevalence and impact of comorbid PTSD, c-PTSD and EUPD on symptom severity in functional neurological disorder: protocol for a systematic review and meta-analysis
Source: BMJ Open. 2025 Oct 16;15(10):e101122. doi: 10.1136/bmjopen-2025-101122 (PMC12530432; doi:10.1136/bmjopen-2025-101122)
Supplement: online supplemental appendix 2 [file bmjopen-15-10-s002.docx]

**Quality assessment form adapted from the JBI checklist for prevalence studies**

| Reviewer: | Date: |
| --- | --- |
| Author: | Year: |

|  |  | Yes/No/Unclear |
| --- | --- | --- |
| Was the sample frame appropriate to address the target population? | [1] Participants were ≥18 years old with a diagnosis of FND |  |
| Were study participants sampled in an appropriate way? | [1] The methods section reports clearly how sampling was performed  [2] convenience samples, such as a street survey or interviewing lots of people at public gathers are not considered to provide a representative sample of the base population |  |
| Was the sample size adequate? | [1] Did the authors conduct a sample size calculation to determine an adequate sample size?  [2] Did the authors consider sample sizes for subgroup (characteristic) analyses?  [3] In large national surveys the study will be large enough whereby a sample size calculation is not required? |  |
| Were the study subjects and setting described in detail? | [1] Certain conditions may vary in prevalence across different geographic regions and populations. Is the study sample described in sufficient detail so that it can be compare to the population of interest? |  |
| Was the data analysis conducted with sufficient coverage of the identified sample? | [1] Did all subgroups of the identified sample respond at a similar rate? |  |
| Were valid methods used for the identification of the condition? | [1] Was FND/ PTSD/ c-PTSD/ EUPD defined as per diagnostic criteria set out in the review’s eligibility criteria?  [2] was symptom severity of core FND symptoms measured using eligible outcome measures? |  |
| Was the condition measured in a standard, reliable way for all participants? | [1] Were those involved in collecting data trained in the use of outcome measures?  [2] was there comparison of results from across the observers?  [3] was the condition measured in the same way for all participants? |  |
| Was there appropriate statistical analysis? | [1] Was the numerator and denominator clearly reported  [2] Were percentages given with confidence intervals?  [3] Did the methods section detail the analytical technique used and how specific variables were measured?  [4] Was the analytical strategy appropriate in terms of the assumptions associated with the approach? For example:  -Continuous – mixed model, ANCOVA  -categorical – mixed model for categorical outcome  -dichotomous – logistic regression |  |
| Was the response rate adequate, and if not, was the low response rate managed appropriately? | [1] Did the authors clearly discuss the response rate and any reasons for non-response and compare persons in the study to those not in the study e.g. in regards to socio-demographic characteristics? |  |

| Overall appraisal: | Include | Exclude | Seek further info |
| --- | --- | --- | --- |

Comments: (including reason for exclusion)
