# Supplementary material for: Prevalence and impact of comorbid PTSD, c-PTSD and EUPD on symptom severity in functional neurological disorder: protocol for a systematic review and meta-analysis
Source: BMJ Open. 2025 Oct 16;15(10):e101122. doi: 10.1136/bmjopen-2025-101122 (PMC12530432; doi:10.1136/bmjopen-2025-101122)
Supplement: Supplementary file 3 [file bmjopen-2025-101122.reviewer_comments.pdf]

PEER REVIEW HISTORY

BMJ Open publishes all reviews undertaken for accepted manuscripts. Reviewers are asked to complete a checklist review form and are provided with free text boxes to elaborate on their assessment. These free text comments are reproduced below.

ARTICLE DETAILS

Title (Provisional)

Prevalence and impact of co-morbid PTSD, c-PTSD and EUPD on symptom severity in Functional Neurological Disorder: protocol for a systematic review and meta-analysis

Authors

Davies, Sian; Rafi, Damir; Rifkin-Zybutz, Raphael; Heyland, Simon; Jadhakhan, Ferozkhan

---

VERSION 1 - REVIEW

**Reviewer**	**1**
**Name**	**Lehn, Alexander**
**Affiliation**	**Princess Alexandra Hospital**
**Date**	**03-Jun-2025**
**COI**	**None**

First of all I will have to declare that I don't have any experience reviewing study protocols for publication. I very much support the publication of study protocols and truly think this should be fostered further and by more journals.

I like the idea of the study and agree with the researchers that this is an important topic. The rationale for the study is outlined well and the objective and methods are sound.

I am surprised that Lea Ludwig's article hasn't been mentioned/referenced (Ludwig, L. et al. Stressful life events and maltreatment in conversion (functional neurological) disorder: systematic review and meta-analysis of case-control studies. The Lancet Psychiatry 5, 307–320 (2018).) I would suggest to review that to ensure there is not too much overlap and/or to see what can be added.

**Reviewer**	**2**
**Name**	**Pick, Susannah**
**Affiliation**	**Institute of Psychiatry Psychology and Neuroscience, Section of Cognitive Neuropsychiatry**

Date **19-Jun-2025**

COI **None**

Thank you for the invitation to review this protocol which proposes a systematic review on PTSD and EUPD in FND. This topic is clinically relevant and the review would be timely and valuable to those interested in the causes, comorbidities and treatment of FND. Whilst the planned review is needed and the methodology generally seems appropriate, the protocol itself could be improved in some areas, particularly by adopting a more succinct writing style, elimination of redundant/repeated content, correction of grammatical errors, and better use of supporting literature. Some specific comments can be found below.

Abstract

•The last two sentences in the introduction section could be phrased more clearly to make apparent the relevance to FND. They could, for example, refer to previous literature showing that rates of cPTSD and/or EUPD traits are higher than comparison groups in FND samples.

•The Methods and Analysis section could be written more succinctly.

Introduction

Overall, this section includes relevant information used to develop the rationale for the review, but the structure requires some work and there is insufficient detail in places - the significant literature on the relationship between trauma and FND is not well explained. For example, there are numerous studies showing that the role of trauma in FND might occur through alterations in brain structure and function, emotional processing, stress reactivity, and heightened dissociation – these lines of evidence could be mentioned. The introduction as it stands does not really provide enough detail on the topic to highlight where exactly the gaps in knowledge are. Many statements in this section are not accompanied by relevant references.

Some specific issues are detailed below.

•Page 4, lines 37-41: Consider citing the original studies that reported on the prevalence of FND in outpatient neurology clinics.

•Page 4, lines 49-52: Consider citing the review by Brown and Reuber 2016. https://pubmed.ncbi.nlm.nih.gov/27084446/

•Page 4, lines 52-53: Please provide at least one reference to support the statement about veterans.

•Page 4, lines 55-56: The Ludwig et al. systematic review on stressful/traumatic events in FND is most relevant to this sentence. https://pubmed.ncbi.nlm.nih.gov/29526521/

•Page 5, lines 3-6: Provide at least one reference to support the statement about MUS.

•Page 5, lines 6-7: The statement that more work needs doing in this area is quite vague – there should be clear, specific problems or knowledge gaps mentioned that the review will aim to address.

•Page 5, lines 13-15: The grammatical structure could be improved in this sentence "we aim to investigate similar with diagnoses that are based around trauma."

•It would be better to present the aims of the review at the end of the introduction, after the background literature and rationale has been presented.

Methods

•Will the authors exclude studies that assessed PTSD using self-report scales such as the PDS-5?

•For FND, will using DSM-5 or ICD-10/11 diagnostic criteria mean that the many studies referring to 'conversion disorder' (or other older terms) would be excluded? These older studies would potentially include diagnoses made with outdated criteria.

•Page 8, Lines 30-32: This is repeated from an earlier section "Studies which involve single case studies or are Randomised controlled trials will be excluded from the systematic review."

•Search strategy – if the review will only include studies that applied diagnostic criteria from DSM-5 or ICD-10/11, it would not be worth searching the databases from inception, as most of the earlier studies would not be eligible for inclusion – please clarify.

•The main search terms and precise eligibility criteria should ideally be included within the manuscript in a box or tables, as these are key aspects of the methodology.

•Data extraction section - There seems to be some repetition of content from the 'Study selection' section here as follows "Each study will be independently screened by two reviewers who will select articles for full-text screening by reading titles and abstracts. The reviewers will apply the aforementioned eligibility criteria in order to determine which articles will be included for review. A third reviewer will be involved in discussion if there are disagreements between the two reviewers about the eligibility of a study."

•PPI – It is not too late to include some PPI in this project, for example, could patient representatives be involved in the searches, data extraction, and/or development of the manuscript? It doesn't seem as though enough consideration has been invested to ensure inclusion of lived experience experts here.

VERSION 1 - AUTHOR RESPONSE

Comments from Reviewer 1
Comment 1: I am surprised that Lea Ludwig's article hasn't been mentioned/referenced (Ludwig, L. et al. Stressful life events and maltreatment in conversion (functional neurological) disorder: systematic review and meta-analysis of case-control studies. The

Lancet Psychiatry 5, 307–320 (2018).) I would suggest to review that to ensure there is not too much overlap and/or to see what can be added.
Response: Thank you for pointing this out. We have reviewed the article by Ludwig, L et al. and included it in our references. We think there is sufficient difference to our proposal which will mean we are adding to the discussion.

Comments from Reviewer 2
Comment 1: Whilst the planned review is needed and the methodology generally seems appropriate, the protocol itself could be improved in some areas, particularly by adopting a more succinct writing style, elimination of redundant/repeated content, correction of grammatical errors, and better use of supporting literature.
Response: We thank you for your comments and have revised the manuscript by paying careful attention to grammatical errors, repeated content and by trying to adopt more succinct prose. We have also incorporated your very helpful suggestions for supporting literature where relevant.

Abstract
Comment 1: The last two sentences in the introduction section could be phrased more clearly to make apparent the relevance to FND. They could, for example, refer to previous literature showing that rates of cPTSD and/or EUPD traits are higher than comparison groups in FND samples.
Response: Thank you for your comment. We have expanded on this introduction section and included relevant references to previous literature to illustrate why ascertaining prevalence rates of PTSD, cPTSD and EUPD is relevant in populations diagnosed with FND. On initial searches there is a scarcity of literature looking at rates of cPTSD in FND samples and hopefully our systematic review will help to explore this in more depth and confirm this. We have referenced previous literature showing that EUPD traits have been noted in FND samples.

Comment 2: The Methods and Analysis section could be written more succinctly.
Response: We have attempted to write this section more succinctly without losing the pertinent information. We have amended the window during which articles from databases will be retrieved from (changed from 'Inception to October 2024' to 'May 1990 to May 2025'). Please see our rationale for this below in our response to comment 4 under the Methods section.

Introduction:
Comment 1: Overall, this section includes relevant information used to develop the rationale for the review, but the structure requires some work and there is insufficient detail in places - the significant literature on the relationship between trauma and FND is not well explained. For example, there are numerous studies showing that the role of trauma in FND might occur through alterations in brain structure and function, emotional processing, stress reactivity, and heightened dissociation – these lines of evidence could be mentioned. The introduction as it stands does not really provide enough detail on the topic to highlight where exactly the gaps in knowledge are. Many statements in this section are not accompanied by relevant references.
Response: Thank you. We have revisited our introduction to ensure that the structure is clearer. We have added details about the neurobiological hypotheses between FND and trauma. We have included additional references to relevant articles.

Comment 2: Page 4, lines 37-41: Consider citing the original studies that reported on the prevalence of FND in outpatient neurology clinics.
Response: (Line 106) We have cited an original study reporting on prevalence of FND in outpatient neurology clinics: Stone, J., Carson, A., Duncan, R., Roberts, R., Warlow, C., Hibberd, C., Coleman, R., Cull, R., Murray, G., Pelosi, A. and Cavanagh, J., 2010. Who is referred to neurology clinics?—the diagnoses made in 3781 new patients. Clinical neurology and neurosurgery, 112(9), pp.747-751.

Comment 3: Page 4, lines 49-52: Consider citing the review by Brown and Reuber 2016. https://pubmed.ncbi.nlm.nih.gov/27084446/
Response: Thank you for this suggestion. We have included this paper in our introduction (line 109)

Comment 4: Page 4, lines 52-53: Please provide at least one reference to support the statement about veterans.
Response: We have included a reference to support this statement (line 117): Salinsky, M., Rutecki, P., Parko, K., Goy, E., Storzbach, D., O'Neil, M., Binder, L. and Joos, S., 2018. Psychiatric comorbidity and traumatic brain injury attribution in patients with psychogenic nonepileptic or epileptic seizures: a multicenter study of US veterans. Epilepsia, 59(10), pp.1945-1953.

Comment 5: Page 4, lines 55-56: The Ludwig et al. systematic review on stressful/traumatic events in FND is most relevant to this sentence. https://pubmed.ncbi.nlm.nih.gov/29526521/
Response: Thank you. We have now included reference to the paper in our introduction.

Comment 6: Page 5, lines 3-6: Provide at least one reference to support the statement about MUS.
Response: Thank you- we have included additional references to support this statement (line 124)

Comment 7: Page 5, lines 6-7: The statement that more work needs doing in this area is quite vague – there should be clear, specific problems or knowledge gaps mentioned that the review will aim to address.
Response: We have amended this section to offer more clarity. By rearranging the order of the introduction and adding supplementary references we hope that we have identified the need for our systematic review. (Lines 125-148)

Comment 8: Page 5, lines 13-15: The grammatical structure could be improved in this sentence "we aim to investigate similar with diagnoses that are based around trauma."
Response: Thank you for this suggestion. We have amended the sentence structure (Line 172)

Comment 9: It would be better to present the aims of the review at the end of the introduction, after the background literature and rationale has been presented.
Response: Yes, we agree with your comment and have rearranged the order of the introduction so that there is a better flow.

Methods:
Comment 1: Will the authors exclude studies that assessed PTSD using self-report scales such as the PDS-5?

Response: Thank you for your comment. We will not be including studies that assess PTSD using self-report scales. Our view aligns with that of Zimmerman (2024) that such scales are screening instruments that should be followed by a diagnostic interview undertaken by a clinician. As our systematic review is attempting to assess prevalence of disorders rather than prevalence of symptoms we feel that it would be more appropriate to use a clinician-administered diagnostic interview rather than scales such as the PDS-5 which assesses symptom severity.
Zimmerman M. The value and limitations of self-administered questionnaires in clinical practice and epidemiological studies. World Psychiatry. 2024 Jun;23(2):210-212. doi: 10.1002/wps.21191. PMID: 38727038; PMCID: PMC11083863.

Comment 2: For FND, will using DSM-5 or ICD-10/11 diagnostic criteria mean that the many studies referring to 'conversion disorder' (or other older terms) would be excluded? These older studies would potentially include diagnoses made with outdated criteria.
Response: We agree with your comment; Using DSM-5 or ICD-10/11 diagnostic criteria will mean that older studies using outdated criteria will be excluded. However our search strategy will still incorporate terms like 'conversion disorder' as many clinicians and publications continue to use such terms when referring to Functional Neurological Disorder. (Lines 270-273)

Comment 3: Page 8, Lines 30-32: This is repeated from an earlier section "Studies which involve single case studies or are Randomised controlled trials will be excluded from the systematic review."
Response: Thank you for highlighting this. We have removed the repeated section.

Comment 4: Search strategy – if the review will only include studies that applied diagnostic criteria from DSM-5 or ICD-10/11, it would not be worth searching the databases from inception, as most of the earlier studies would not be eligible for inclusion – please clarify.
Response: We agree with your comment and since the DSM-5 was published 27th May 2013 and the ICD-10 was first endorsed in May 1990 we have adjusted the search window from May 1990 to May 2025. May 2025 has been chosen as this was the date of the most recent search that we undertook, however we will update this prior to the publication of the final manuscript outlining the findings from the systematic review. (Lines 270-273)

Comment 5: The main search terms and precise eligibility criteria should ideally be included within the manuscript in a box or tables, as these are key aspects of the methodology.
Response: Thank you. We have included the main search terms and eligibility criteria in the manuscript. As a consequence we have removed supplementary appendix 1 and re-numbered the supplementary documents to reflect this change. (Line 279 and Line 302)

Comment 6: Data extraction section - There seems to be some repetition of content from the 'Study selection' section here as follows "Each study will be independently screened by two reviewers who will select articles for full-text screening by reading titles and abstracts. The reviewers will apply the aforementioned eligibility criteria in order to determine which articles will be included for review. A third reviewer will be involved in discussion if there are disagreements between the two reviewers about the eligibility of a study."
Response: Thank you for pointing this out to us. We have removed the repeated content.

Comment 7: PPI – It is not too late to include some PPI in this project, for example, could patient representatives be involved in the searches, data extraction, and/or development of the

manuscript? It doesn’t seem as though enough consideration has been invested to ensure inclusion of lived experience experts here.
Response: Thank you for your valuable suggestion regarding Patient and Public Involvement (PPI). We agree on the critical importance of incorporating lived experience to enrich research and ensure its relevance.
For this specific systematic review, given its technical methodology involving precise database searches and structured data extraction, direct involvement of patient representatives in these particular stages presents practical challenges. However, we are committed to ensuring that the insights gained from this review where relevant will directly inform future research on clinical initiatives where patient and public involvement will be more integral, particularly in developing patient-centred recommendations. We appreciate your emphasis on this vital aspect of research.

Thank you very much for considering our resubmission.

VERSION 2 - REVIEW

Reviewer 2

Name Pick, Susannah

Affiliation Institute of Psychiatry Psychology and Neuroscience, Section of Cognitive Neuropsychiatry

Date 15-Sep-2025

COI

Thank you for addressing my comments.
